# Traditional japanese diet score and the sustainable development goals by a global comparative ecological study

**DOI:** 10.1186/s12937-024-00936-2

**Published:** 2024-03-21

**Authors:** Tomoko Imai, Keiko Miyamoto, Ayako Sezaki, Fumiya Kawase, Yoshiro Shirai, Chisato Abe, Masayo Sanada, Ayaka Inden, Norie Sugihara, Toshie Honda, Yuta Sumikama, Saya Nosaka, Hiroshi Shimokata

**Affiliations:** 1 Department of Food Science and Nutrition, Doshisha Women’s College of Liberal Arts, Kyoto, Japan; 2 Institute of Health and Nutrition, Nagoya University of Arts and Sciences, Aichi, Japan; 3 Department of Nursing, Nagoya University of Arts and Sciences, Aichi, Japan; 4 National Cancer Center Japan, Tokyo, Japan; 5 Department of Nutrition, Asuke Hospital Aichi Prefectural Welfare Federation of Agricultural Cooperatives, Aichi, Japan; 6 Graduate School of Nutritional Science, Nagoya University of Arts and Sciences, Aichi, Japan; 7 Persuasive Technology Group, Life Science Laboratories, KDDI Research, Inc, Fujimino, Japan; 8 Department of Food and Nutrition, Tsu City College, Mie, Japan; 9 Department of Nursing, Heisei College of Health Sciences, Gifu, Japan; 10 Clinical Nutrition Unit, Hamamatsu University Hospital, Shizuoka, Japan; 11 Faculty of Health and Social Services, Kanagawa University of Human Services, Kanagawa, Japan; 12 Department of Nutrition, Japanese Red Cross Aichi Medical Center Nagoya Daiichi Hospital, Aichi, Japan

**Keywords:** Dietary pattern, Japanese diet, SDGs, GHG, Ecological study

## Abstract

**Background:**

Reducing the environmental impact of the food supply is important for achieving Sustainable Development Goals (SDGs) worldwide. Previously, we developed the Traditional Japanese Diet Score (TJDS) and reported in a global ecological study that the Japanese diet is associated with reducing obesity and extending healthy life expectancy etc. We then examined the relationship between the TJDS and environmental indicators.

**Methods:**

The average food (g/day/capita) and energy supplies (kcal/day/capita) by country were obtained from the Food and Agriculture Organization of the United Nations Statistics Division database. The TJDS was calculated from eight food groups (beneficial food components in the Japanese diet: rice, fish, soybeans, vegetables, and eggs; food components that are relatively unused in the traditional Japanese diet: wheat, milk, and red meat) by country using tertiles, and calculated the total score from − 8 to 8, with higher scores meaning greater adherence to the TJDS. We used Land Use (m^2^), Greenhouse gas (GHG) emissions 2007/2013 (kg CO_2_eq), Acidifying emissions (g SO_2_eq), Eutrophying emissions (g PO_4_^3−^ eq), Freshwater (L), and water use (L) per food weight by Poore et al. as the environmental indicators and multiplied these indicators by each country’s average food supply. We evaluated the cross-sectional and longitudinal associations between the TJDS and environmental indicators from 2010 to 2020. This study included 151 countries with populations ≥ 1 million.

**Results:**

Land use (β ± standard error; -0.623 ± 0.161, *p* < 0.001), GHG 2007 (-0.149 ± 0.057, *p* < 0.05), GHG 2013 (-0.183 ± 0.066, *p* < 0.01), Acidifying (-1.111 ± 0.369, *p* < 0.01), and Water use (-405.903 ± 101.416, *p* < 0.001) were negatively associated with TJDS, and Freshwater (45.116 ± 7.866, *p* < 0.001) was positively associated with TJDS after controlling for energy supply and latitude in 2010. In the longitudinal analysis, Land Use (β ± standard error; -0.116 ± 0.027, *p* < 0.001), GHG 2007 (-0.040 ± 0.010, *p* < 0.001), GHG 2013 (-0.048 ± 0.011, *p* < 0.001), Acidifying (-0.280 ± 0.064, *p* < 0.001), Eutrophying (-0.132 ± 0.062, *p* < 0.05), and Water use (-118.246 ± 22.826, *p* < 0.001) were negatively associated with TJDS after controlling for confounders.

**Conclusions:**

This ecological study suggests that the traditional Japanese dietary pattern might improve SDGs except Fresh water.

**Supplementary Information:**

The online version contains supplementary material available at 10.1186/s12937-024-00936-2.

## Introduction

The adoption of the 2030 Agenda for Sustainable Development at the United Nations Summit in 2015 [1] included 17 Sustainable Development Goals (SDGs). Reducing the environmental impact of diet is essential for achieving the SDGs worldwide. The Food and Agriculture Organization (FAO) and the United Nations World Health Organization (WHO) published sustainable healthy diet guiding principles in Rome in 2019 [2]. In the same year, Willet et al. [3] also reported a sustainable healthy diet called the EAT-Lancet Commission. Nelsom et al. [4] conducted a systematic review and reported that adherence to well-balanced dietary patterns promotes better health and has a lower negative impact on environmental outcomes such as greenhouse gas (GHG) emissions, the environmental outcomes assessed vary widely depending on the study. Many studies have examined single indicators, such as GHGs, and only a few have examined a combination of environmental indicators of different quality. The review also majority included US and European studies with very few global studies and no Japanese studies. Sugimoto et al. [5] studied diet-related GHGs and major food contributors in Japan using the original GHG database. Few studies have examined the relationship between Japanese diet and environmental indicators [6, 7]. However, studies on the relationship between a healthy diet and environmental outcomes are still limited not only for the Japanese diet but also for the Healthy Eating Index and the Mediterranean Diet Score [8].

According to the WHO data [9], Japan has one of the lowest obesity rates and one of the healthiest and longest life expectancies globally. After Japanese dietary culture, known as ‘WASHOKU’ was registered as a United Nations Educational, Scientific, and Cultural Organisation intangible cultural heritage in 2012, the appeal of the Japanese diet was promoted at the national level [10]. Kobayashi et al. [11] reported from the Japan Public Health Center-based Prospective Study (JPHC) that the greater the diversity of foods, including fruit and soy consumed, the lower the total mortality and mortality from major diseases. Many studies have reported that rice, fish, and soy products are effective in preventing obesity [12], ischaemic heart disease [13–15], and other diseases [15–19]. A Japanese dietary pattern high in these foods may be beneficial for health. For example, Kurotani et al. scored the Japanese diet according to the Japanese Food Guide [18] and reported that the higher the adherence to the food guide, the lower the total mortality rate in JPHC. Several Japanese diet scores have been developed, and the health benefits of Japanese dietary patterns have been reported [20–24]. However, none of the studies have examined the usefulness of the Japanese diet using data from multiple countries. This may be because the published Japanese diet score is designed to count dishes unique to Japanese cuisines, such as staple and main dishes [18, 20–22], pickles, and miso soup [17, 23, 24]. Therefore, we previously developed the Traditional Japanese Diet Score (TJDS) and reported in a global ecological study that the Japanese diet is associated with reducing obesity, incidence of ischaemic heart disease, and extending healthy life expectancy [25]. Subsequently, we reported that the TJDS contributed to breast cancer prevention [26] and lowered all-cause mortality [27], and suicide prevention [28].

Contrarily, Poore et al. [29] reported on the relationship between food supply and environment by creating seven environmental indicators [(Land use, GHG Emissions 2007 (GHG2007) and 2013 (GHG2013), Acidifying Emissions (Acidifying), Eutrophying Emissions (Eutrophying), Freshwater Withdrawals (Freshwater), and stress-weighted Water use (Water use)], covering food production, processing, and retailing from open data on the environmental impacts of 38 700 farms, and 1600 processors, packaging types, and retailers, and their systematic review. The dataset covers 90% of global protein and calorie consumption. Poore et al. stated that these indicators could explain 80% of the Food and Agriculture Organization of the United Nations Statistics Division database (FAOSTAT), which shows the food supply in each country. Using the data developed by Poore et al. it was possible to simultaneously examine the relationship between diet and seven environmental indicators of different quality. Furthermore, since Japanese is monoracial country, a large variance in the Japanese food score using only Japanese data was not expected. Therefore, we conducted a cross-sectional and longitudinal ecological study to examine whether the Japanese diet contributes to sustainability using the TJDS and the environmental indicators of Poore et al. [29].

By conducting these analyses, this study could contribute to the achievement of eight major SDGs goals (2; zero hunger, 3; good health and well-being, 6; clean water and sanitation, 11; sustainable cities and communities, 12; responsible consumption and production, 13; climate action, 14; life bellow water, and 15; life on land).

## Methods

### Variables

#### Foods

The FAOSTAT provides food and agricultural data for over 245 countries and regions. It covers all FAO regional groupings from 1961 to 2020 [30]. These data include the average food supply per capita per day (g/day/capita) and energy supply (kcal/day/capita) by country, excluding losses between production and households. Due to a change in the aggregation method [31], we used the supply of foods examined from 2010 to 2020. Before starting the ecological study, the FAOSTAT was used to compare the food supply and intake using Japanese data, and the difference between the two was found to be acceptable [32].

#### Traditional Japanese Diet score (TJDS)

The TJDS was calculated in reference to the Mediterranean diet score proposed by Trichopoulou et al. [33]. The FAOSTAT does not have detailed nutrient or food data; only supplies of major food groups are available. Hence, the study by Trichopoulou et al. was referred to since their scoring is of a typical Mediterranean diet and uses common food groups rather than saturated fatty acids and added sugar beverages. Originally, TJDS was calculated using nine indicated food components characteristic of the Japanese diet (beneficial food components in the Japanese diet, rice, fish, soybeans, vegetables, eggs, and seaweeds, food components that are relatively unused in traditional Japanese diet, wheat, milk, and red meat). Each of the nine food components was divided into tertiles in order of the food supply of 1000 kcal per capita. The six beneficial food components were given 1, 0, and − 1 points in order of the food supply, and the three relatively unused food components were given − 1, 0, and 1 points. The total score ranged from − 9 to 9; higher scores meant greater adherence to a traditional Japanese diet. We have previously reported associations between the TJDS and several diseases [25–28]. However, after closely examining the food supply of each country in the FAOSTAT, we modified the TJDS to include only eight food components and excluded seaweed.since only a few countries have seaweed as a part of their diet. This paper used a modified TJDS score from − 8 to 8, which was calculated from 8 food groups. The details are described in a previous study [27].A list of foods that are included in the TJDS and the FAOSTAT is shown in Appendix Supplementary Table S1.

#### Environmental indicators

We used the Supplemental Data from Poore et al. [29]. Each indicator value was expressed as 1 functional unit (FU) for 43 food groups. The environmental indicators included Land use (m^2^/FU), GHG2007 and GHG2013 (kg CO_2_eq/FU, based on the Intergovernmental Panel on Climate Change (IPCC) 2007 or 2013), Acidifying Emissions (g SO_2_ eq/FU), Eutrophying Emissions (g PO_4_^3−^eq/FU), Freshwater Withdrawals (L/FU), and stress-weighted Water use (L/FU). Land use included temporary or permanent seed or cultivated land and pasture, and GHG2007 or GHG2013 included CO_2_, CH_4_, and N_2_O air emissions from the 2007 or 2013 IPCC report [34]. Acidifying Emissions indicate the amount of SO_2_, NH_3_, and NO_x_ released into the air; Eutrophying Emissions indicate the amount of NH_3_ and NO_x_ released into the air and No_3_^−^, NH_4_^+^, P, and N released into the water. Freshwater Withdrawals represent the amount of water used for irrigation, drinking, ponds, and processing. Stress-weighted Water indicates scarcity-weighted Freshwater Withdrawals. For more information, please refer to the supplement data of Poor et al. [29]. The value of 1 FU depends on the food group; for example, 1 FU of rice is equivalent to 1 kg of full-grain white or brown rice. The value of these indicators was calculated by multiplying the supply of each food group equivalent to 1 FU determined by the FAOSTAT for each indicator’s median amount value in 1 FU, to reduce the effect of outliers. The total indicator value in the 43 food groups was used as the total of each indicator’s value.　A list of foods that are included in the Poores et al. for the SDGs in their study and the FAOSTAT is shown in Supplementary Table S2.

#### Co-variables

Energy supply data were obtained from the FAOSTAT. Agriculture is affected by climate, and the absolute latitude values for the centre of each country were obtained from the covariate database of the Global Burden of Diseases, Injuries, and Risk Factors Study 2019 database (GBD 2019) [35]. The total population (population) was identified using the World Bank database 2021 [36].

### Statistical analysis

A total of 151 countries with populations of more than 1 million, for which all data were available, were used for the analysis. To examine the distribution and overtime change of theTJDS, environmental indicators, and co-variables, the mean value of each variable in 2010, 2015, and 2020 was tested using analysis of variance, and the trend was tested using a general linear model. Cross-sectional relationships between the TJDS and environmental indicators were evaluated using a general linear regression model controlled for energy and latitude in 2010, 2015, and 2020. We controlled for energy to examine the relative impact of TJDS on energy and latitude because we wanted to exclude the effect of climate. Since controlling for country-specific socioeconomic variables and lifestyle differences as covariates would have changed the relationship between the environmental index and the Japanese food score, we did not control for these variables. Based on the GBD classification, the countries were divided into seven super regions (Supplementary Table S3), and the association between the TJDS and environmental indicators was shown by raw value bubble plotting with the population of countries as the bubble size (regression lines if there was a significant association) in 2020. We further analysed the longitudinal association between the TJDS and environmental indicators and TJDS and the interaction with year between 2010 and 2020, controlling for energy and latitude using linear mixed models. The models were fitted by maximising the log-likelihood. All variables, except the environmental indicators, were centralised in the analysis. All analyses were performed using R version 4.3.1 [37], and the generalised linear mixed-effects model was fitted using the ‘lme’ function of the ‘nlme’ package [38]. P values < 0.05 were considered statistically significant.

## Results

The TJDS, environmental indicators, and co-variables from 2010 to 2020 are listed in Table 1 for each of the three years. Neither the TJDS nor the environmental indicators changed significantly over the 10-year period. The mean ± SD of theTJDS and the environmental indicators in 2010 were − 0.4 ± 2.8 (TJDS), 10.6 ± 6.1 m^2^ (Land use), 5.4 ± 2.6 kg CO_2_ eq (GHG2007), 6.0 ± 2.9 kg CO_2_ eq (GHG2013), 39.2 ± 20.4 g SO_2_ eq (Acidifying), 33.5 ± 16.8 g PO_4_^3−^ eq (Eutrophying), 587 ± 284 L (Freshwater), and 12,056 ± 7786 L (Freshwater).

**Table 1:** Traditional Japanese Diet Score and the characteristics of countries in 2010, 2015, and 2020

Year	2010 (*n*=145)		2015 (*n*=148)		2020 (*n*=151)		p value	
	Mean	SD^a^	Mean	SD^a^	Mean	SD^a^	ANOVA	trend^b^
Land use (m^2^)	10.6	6.1	10.5	6.0	10.5	6.2	0.976	0.914
GHG2007^c^ (kg CO_2_ eq)	5.4	2.6	5.4	2.6	5.5	2.6	0.964	0.803
GHG2013^d^ (kg CO_2_ eq)	6.0	2.9	6.0	2.9	6.1	3.0	0.973	0.834
Acidifying (g SO_2 eq_)	39.2	20.4	39.5	20.2	40.2	20.6	0.919	0.687
Eutrophying (g PO_4_^3-^ eq )	33.5	16.8	33.5	16.2	33.8	16.6	0.979	0.861
Fresh water^e^ (L)	587	284	591	285	601	286	0.909	0.673
Water use^f^ (L)	12,056	7786	12,297	7933	12,518	8019	0.882	0.616
Population (milion)	47	155	49	161	51	165	0.977	0.829
Energy supply (1,000 kcal/capital/day)	1035	363	1059	381	1070	390	0.726	0.432
TJDS^g^	-0.4	2.8	-0.3	2.8	-0.1	2.8	0.703	0.416

a: Standard deviation,b: general linear model,

b: general linear model,

c: greenhouse gas emissions 2007

d: greenhouse gas emissions 2013

f: Stress-weighted Water use

g: Traditional Japanese Diet Score

Table 2 shows the cross-sectional analysis results of theTJDS and environmental indicators for 2010, 2015, and 2020. Land use (β ± standard error; -0.623 ± 0.161, *p* < 0.001), GHG2007 (-0.149 ± 0.057, *p* < 0.05), GHG2013 (-0.183 ± 0.066, *p* < 0.01), Acidifying (-1.111 ± 0.369, *p* < 0.01), and Water use (-405.903 ± 101.416, *p* < 0.001) were negatively associated with the TJDS in 2010. While Freshwater (45.116 ± 7.866, *p* < 0.001) was positively associated with TJDS. However, no significant association was found between the TJDS and Eutrophying (0.278 ± 0.396, n.s.). The cross-sectional results for 2015 and 2020 showed the same trend, although the degree of significance varied.

**Table 2:** Partial regression coefficients of the Traditional Japanese Diet Score in 2010, 2015, and 2020

Year	2010 (*n*=145)^a^			2015 (*n*=148)^a^			2020 (*n*=151)^a^		
	β	SE^b^		β	SE^b^		β	SE^b^	
Land use	-0.623	0.161	***	-0.673	0.155	***	-0.598	0.163	***
GHG2007^c^	-0.149	0.057	*	-0.148	0.056	**	-0.128	0.057	*
GHG2013^d^	-0.183	0.066	**	-0.184	0.065	**	-0.163	0.065	*
Acidifying	-1.111	0.369	**	-0.991	0.368	**	-0.876	0.367	*
Eutrophying	0.278	0.396		0.375	0.391		0.477	0.386	
Fresh water^e^	45.116	7.866	***	49.144	7.412	***	48.156	7.365	***
Water use^f^	-405.903	101.416	***	-326.073	105.280	**	-345.517	104.617	**

a: All models were controlled for energy supply and absolute value of latitude

b: standard error, *** *p* < 0.001; ** *p* < 0.01; * *p* < 0.05,

c: greenhouse gas emissions 2007

d: greenhouse gas emissions 2013

e: Freshwater withdrawals

f: Stress-weighted Water use

The 10-year longitudinal analysis of the TJDS and environmental indicators from 2010 to 2020, controlling for co-variables, is shown in Table 3. The TJDS was significantly negatively associated with Land use (β ± standard error; -0.116 ± 0.027, *p* < 0.001), GHG2007 (-0.040 ± 0.010, *p* < 0.001), GHG2013 (-0.048 ± 0.011, *p* < 0.001), Acidifying (-0.280 ± 0.064, *p* < 0.001), Eutrophying (-0.132 ± 0.062, *p* < 0.05), and Water use (-118.246 ± 22.826, *p* < 0.001); however, there was no significant association with Freshwater (0.858 ± 0.793, n.s.). The analysis showed no significant difference between the TJDS and year, even after controlling for co-variates, except Freshwater and Water use. The model without interactions had a better fit for almost all environmental indicators.

**Table 3:** Fixed effects of Traditional Japanese Diet Score and interaction between Traditional Japanese Diet Score and year in linear mixed model^a^

	TJDS^b^			TJDS × year^c^		
	β	SE^d^		β	SE^d^	
Land use	-0.116	0.027	***	-0.005	0.006	
GHG2007^e^	-0.040	0.010	***	-0.001	0.002	
GHG2013^f^	-0.048	0.011	***	-0.002	0.002	
Acidifying	-0.280	0.064	***	-0.006	0.011	
Eutrophying	-0.132	0.062	*	0.005	0.011	
Fresh water^g^	0.858	0.793		-0.284	0.136 *	
Water use^h^	-118.246	22.826	***	-7.868	3.913 *	

a: This models was controlled for energy supply, year, and absolute value of latitude

b: Traditional Japanese Diet Score

c: TJDS year interaction

d: standard error, *** *p* < 0.001; ** *p* < 0.01; * *p* < 0.05

e: greenhouse gas emissions 2007

f: greenhouse gas emissions 2013

g: Freshwater withdrawals

h: Stress-weighted Water use

**Fig. 1:**
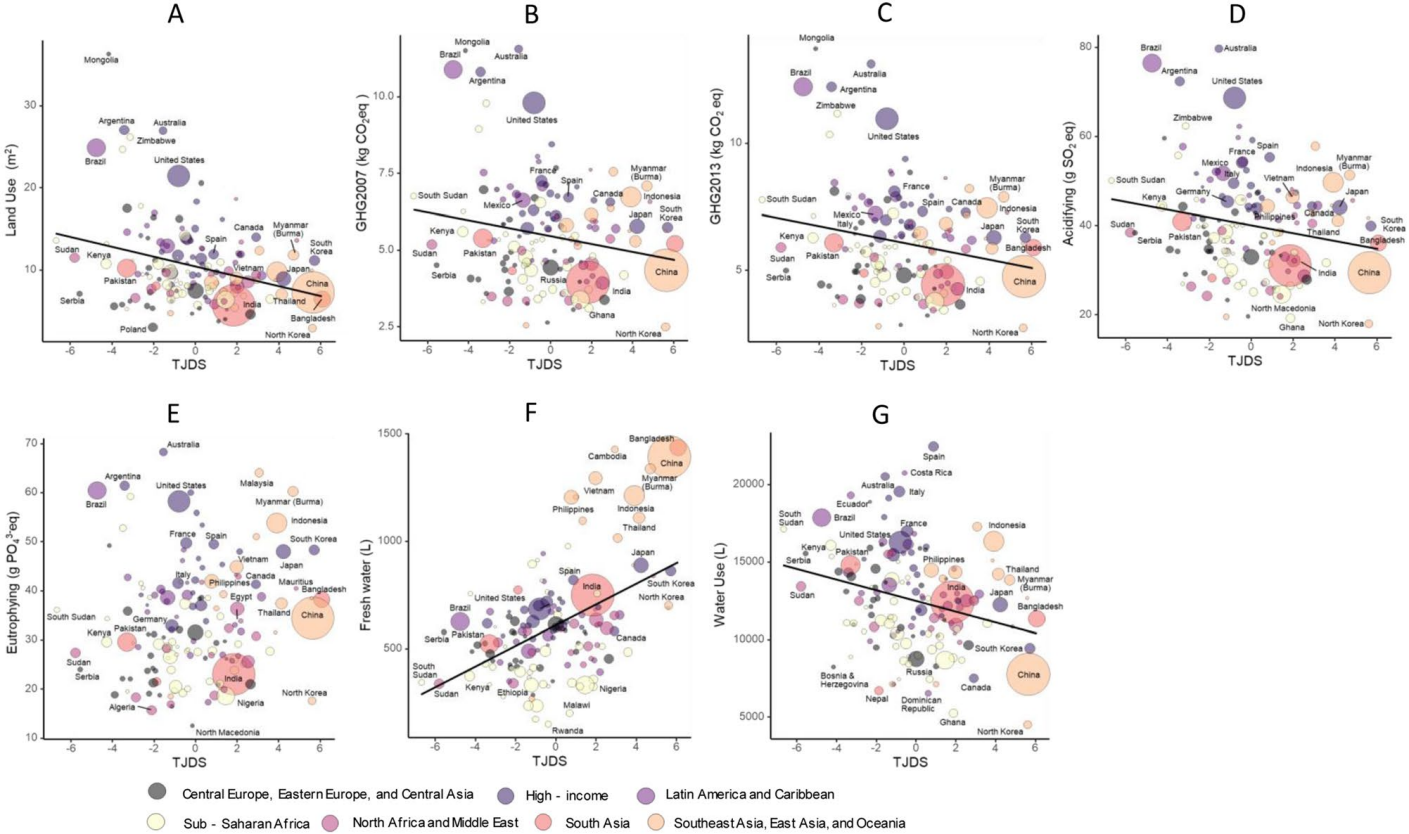
The relationships between TJDS and environmental indicators prevalence in 2020. The figures show the relationship between the TJDS and environmental indicators in 2020, respectively. **A**: Land use (m^2^/FU), **B** Greenhouse gas (GHG) Emissions 2007 (kg CO_2_eq), **C** Greenhouse gas (GHG) Emissions 2013 (kg CO_2_eq) **D** Acidifying Emissions (g SO_2_ eq), **E** Eutrophying Emissions (g PO_4_^3-^eq), **F** Freshwater Withdrawals (L), and **G** stress-weighted Water use (L). The countries were divided into seven Super Regions based on GBD classification, and the population-weighted TJDS (horizontal) and environmental indicators (vertical) were shown by raw value plotting (with regression lines if there was a significant association) in 2020

The relationships between the population-weighted TJDS and the seven environmental indicators prevalence by super-regions of GBD classification in 2020 are shown in Fig. 1. Except for Eutrophying, there were significant associations between the TJDS and environmental indicators; however, the variation in the position of the plots in each country differed. For example, the United States had large residues, except for Freshwater, whereas China had large Freshwater residues. Conversely, some countries, such as Japan and South Korea, have low residues for all environmental indicators.The TJDS tended to be lower in Europe region and higher in South Asia, Southeast Asia, and East Asia. However, residues differed by country and type of environmental indicator.

## Discussion

We examined the cross-sectional and longitudinal associations between the TJDS and seven environmental indicators using Poore et al.’s supplemental data [29]. The association between the TJDS and environmental indicators was similar in 2010, 2015, and 2020. The longitudinal analysis showed the same trends for the five environmental indicators as the cross-sectional analysis. The TJDS was negatively associated with land use, GHG2007, GHG2013, Acidifying, and Water use in cross-sectional and longitudinal analyses. While Eutrophying (cross-sectional, n. s.; longitudinal, negative) and Freshwater (cross-sectional, positive longitudinal, n.s.) had different results in the cross-sectional and longitudinal analyses. These results suggest that the negative associations between TJDS and Land use, GHG, Acidifying, and Water use are not incidental but permanent.

This is the first study to examine the relationship between Japanese-style diet and eight of the 17 SDGs on a longitudinal and global scale. Using the original GHG database, Sugimoto et al. [5] reported that the top contributor to GHGs was meat, followed by fish and seafood. Oita et al. [7] examined the changes in the nitrogen footprint (NF) of the Japanese diet from 1961 to 2011 using FAOSTAT. They reported that the protein intake in 1975 was the closest to the protein recommendation and reported a lower NF than that in 2011. The protein intake percentage from meat increased from 3 to 15%, while that from rice decreased from 45 to 28% during this period in Japan. However, we did not find any studies examining the relationship between Japanese-type diets and environmental indicators in other countries, including Japan. Nelson et al. [4] reported that plant-based foods, such as dietary guideline-related diets, Mediterranean-style diets, the Dietary Approaches to Stop Hypertension diet, and other sustainable diet scenarios, were lower in total energy and had less impact on environmental indicators such as GHG and land use than animal-based foods from 23 articles extracted through a systematic review. Other systematic reviews have reported similar results [39]. The relationship between the TJDS and the seven environmental indicators we used were consistent with that reported in a previous report, indicating that the Japanese diet is likely to have a small environmental impact on a global scale in Land use GHG, Acidifying, and Water use not only TJDS is useful for health. The Japanese diet is also consumed worldwide [10] and is considered to be internationalized. The results suggest that the Japanese diet is also a healthy dietary option. For Asian countries where rice is the staple food among cereals, it is a significant finding that a rice-based diet was found to be negatively associated with several environmental indicators.

The problem with the Japanese diet regarding its environmental impact is that rice cultivation requires a large amount of freshwater [40, 41]. Our cross-sectional results also show a positive correlation between the TJDS and Freshwater use. Islam et al. [42] reported that more than half of the world’s population consumes rice; however, rice production systems are the largest anthropogenic wetlands. Several agronomic strategies have been proposed to improve water-use efficiency and reduce GHG emissions. They concluded that improved water management and timely coordination of N fertiliser with crop demand could reduce water use and N loss via N_2_O and CH_4_ emissions. Our longitudinal analysis, including the interaction between the TJDS and year, showed that the association between the TJDS and freshwater was reversed in 2018, and although not significant, the higher the TJDS, the less freshwater (data not presented). These results may explain why the Freshwater and　Eutrophying　analyses differed between the cross-sectional and longitudinal analyses in our study.

Our data also showed that even countries with very similar TJDS scores have different residuals for environmental indicators. Although the scores were similar, the food supply that made up the scores differed greatly, which may have contributed to the variation in the magnitude of the effects of the environmental indicators.

In addition, the mean scores of TJDS were − 0.4 in 2010 and − 0.1 in 2020; however, our analysis showed no period change in scores between 2010 and 2020. Economic development and changes in eating habits have been reported to alter environmental impact [7, 29, 40–42]. In our data, we compared China, India, and Japan. GHG2013 in 2010 was 11.4 kg CO_2_ eq in China, 2.8 kg CO_2_ eq in India, and 5.9 kg CO_2_ eq in Japan, a global total of 871.2 kg CO_2_ eq; in 2020, it was 13.0 kg CO_2_ eq in China, 3.3 kg CO_2_ eq in India, and 6.1 kg CO_2_ eq in Japan, a global total of 918.0 kg CO_2_ eq, the same was true for GHG2007. Similarly, Land use and Acidifying were increasing, with global totals of 1535.7 m^2^ (in 2010), 1587.7 m^2^ (in 2020), 5671.2 g SO_2_ eq (in 2010), 6049.9 g SO_2_ eq (in 2020), respectively. Eutrophying and Freshwater trends vary by country; however, the total was increasing. Aleksandrowicz et al. reported that in India, where malnutrition is still prevalent, the spread of healthier diets could result in a slight increase in the environmental footprint of the food system over the current situation. However, an even larger increase is expected if the diets consumed by the wealthiest people become widespread [43]. Natori et al. [44] examined the possibility of using the Satoyama Index, which was developed with a focus on biodiversity and tested in Japan for socio-ecological production landscape mapping on a global scale. It can be used globally to identify landscapes resulting from complex interactions between people and nature, with statistical significance. International comparisons using the same indicators may create an environment where environmentally friendly agricultural technologies can be provided. Japanese-style agriculture will also be helpful in achieving SDGs.

The SDGs must be achieved not only from the production and distribution aspects but also from the consumer aspect. From the consumer perspective, obesity prevention and food loss reduction are major issues that can be addressed to achieve the SDGs [40, 45]. However, using the TJDS and environmental indicators of Poore et al., we were able to show that a traditional Japanese-style diet was environmentally friendly. Some have reported a disconnect between current public health and SDGs studies [40]. Although the SDGs are diverse, Poore et al. pointed out that environmental impacts need to be judged comprehensively and that it is necessary to study all aspects of food production, distribution, and retail to consumers and create measures starting with items that are easy to address. This study may also help in this regard.

The strength of this study is that we examined the relationship between Japanese-style diets and multiple environmental indicators on a global scale and longitudinally considering the environmental impacts from production to consumers. Agricultural producers and consumers in each country can compare the seven environmental indicators internationally and choose the indicators most likely to help them achieve SDGs.

Conversely, a limitation of this study is whether these environmental indicators reflect changes in environmental impacts over the past decade. This study used large amounts of open data and their systematic reviews to create seven environmental indicators. The data were centred on 2010, and the external data related to 2009-11 [29]. Poore et al’s efforts the data collected for the review were biased, and some areas were inferred and supplemented with outside information. Moreover, foods in the FAOSTAT that were included from Poore et al.’s supplement data in this paper do not cover all food items in the FAOSTAT.

Despite a few problems, no other environmental indicators similar to this one was found.

Another limitation is that this was an ecological study. In the future, we would like to examine whether these environmental indicators can be used to explain the relationship between an individual’s diet and environmental factors using cohort data or other methods.

In conclusion, TJDS is an indicator of the association between Japanese diet and health events [16, 25–28]. In addition, TJDS was negatively related to land use, GHG emissions, Acidifying emissions, and stress-weighted Water use. Japanese style diet could contribute to the SDGs on a global scale.

### Electronic supplementary material

Below is the link to the electronic supplementary material.

Supplementary Material 1

## Data Availability

The data associated with this paper are available from the organizations listed in the text or from the corresponding author upon reasonable request.

## Abbreviations

SDGs: Sustainable Development Goals
TJDS: Traditional Japanese Diet Score
GHG: Greenhouse Gas
WHO: the United Nations World Health Organization
FAO: The Food and Agriculture Organization
Acidifying: Acidifying Emissions
Eutrophying: Eutrophying Emissions
Freshwater: Freshwater Withdrawals
Water use: stress-weighted Water use
FAOSTAT: Food and Agriculture Organization of the United Nations Statistics Division database
JPHC: Japan Public Health Center-based Prospective Study
IPCC: Intergovernmental Panel on Climate Change
FU: functional unit
GBD: Global Burden of Diseases, Injuries, and Risk Factors Study
